# Smoke-Free Ordinances and Policies Protect Youth, but Ordinances Appear to Have Little Impact on Non-Combustible Tobacco Use

**DOI:** 10.3390/children6030044

**Published:** 2019-03-11

**Authors:** Nell Valentine, Emily McClelland, Robert McMillen

**Affiliations:** 1 Mississippi State University, Mississippi State, MS 39759, USA; nell.valentine@ssrc.msstate.edu (N.V.); emily.mcclelland@ssrc.msstate.edu (E.M.); 2 American Academy of Pediatrics Julius B. Richmond Center of Excellence, Elk Grove Village, IL 60143, USA

**Keywords:** smoke-free, tobacco, youth, prevalence, combustible tobacco products, e-cigarette

## Abstract

Smoke-free ordinances and policies protect youth from exposure to secondhand smoke (SHS) and cigarette use. This study investigated whether smoke-free ordinances also protect youth from the use of other tobacco products. We compared the prevalence of SHS exposure, cigarette smoking, cigar smoking, smokeless tobacco use, and e-cigarette use among high school students living in a municipality with or without a smoke-free ordinance and in homes with and without smoke-free policies. Data were analyzed using the 2017 Mississippi Youth Tobacco Survey (*n* = 1923). Smoke-free ordinances were found to be associated with lower prevalence of SHS exposure (41.9% vs. 51.5%), cigarette smoking (5.1% vs. 11.4%), and cigar smoking (7.2% vs. 10.9%). There were no differences in smokeless tobacco use (6.6% vs. 6.5%) or e-cigarette use (11.2% vs 12.1%). Smoke-free homes were associated with lower prevalence of SHS exposure (38.0% vs 74.6%), cigarette smoking (4.8% vs. 17.6%), cigar smoking (6.4% vs. 16.4%), smokeless tobacco use (4.9% vs. 13.2%), and e-cigarette use (9.6% vs. 19.5%), *p* < 0.05 for all comparisons. The results suggest that smoke-free ordinances and policies protect against exposure to tobacco smoke and use of combustible tobacco products, but smoke-free ordinances do not protect from smokeless tobacco and e-cigarette use. Tobacco-free, rather than smoke-free, ordinances might offer more protection.

## 1. Introduction

Tobacco is the leading cause of preventable death in the United States [1]. Although most of the mortality attributable to tobacco occurs among adults, children’s passive tobacco smoke exposure harms their health during childhood and adulthood. These harms include higher rates of sudden infant death syndrome, asthma prevalence and severity, lower respiratory infections, otitis media, and lung cancer as adults, as well as deleterious effects on behavior and cognition [2,3,4,5].

In recognition of these harms, 25 states, and the District of Columbia, have implemented statewide smoke-free legislation for indoor public places, including restaurants, bars, and workplaces [6]. In private domains, more than eight in ten U.S. households do not allow anyone to smoke inside the home [7]. These restrictions were initially enacted to protect nonsmokers from the harms of secondhand smoke (SHS) exposure. Subsequent research illustrated another impact, these restrictions were also found to be associated with decreased prevalence of cigarette smoking, established smoking, and lower daily cigarette consumption [8,9,10,11,12,13,14,15,16,17,18,19,20,21,22,23,24].

Restrictions on smoking appear to reduce smoking via two routes. First, smoke-free policies interrupt and inconvenience smoking behaviors. Following the implementation of smoke-free policies, smokers may quit or reduce consumption due to the inconvenience of leaving a venue in order to go to a place where smoking is permitted [16,19]. Second, smoke-free policies influence the social climate impacting decisions about smoking. Restrictions that limit cigarette smoking and emphasize the rights of nonsmokers may also change societal and community norms to be more unfavorable towards smoking behaviors [25,26,27,28].

Smoke-free local ordinances [10] and household policies [29,30,31] protect youth from exposure to tobacco smoke and cigarette use. This study investigated whether they were also protected from the use of other tobacco products. We compared the prevalence of cigarette smoking, cigar smoking, smokeless tobacco (SLT) use, and e-cigarette use among Mississippi high school students living in a municipality with or without a smoke-free ordinance; and in homes with and without smoke-free policies. Overall, the use of these tobacco/nicotine products in Mississippi youth were found to be similar. There was a statistical difference in past 30-day prevalence of cigarette smoking (7.2%), cigar smoking (8.4%), smokeless tobacco (6.6%), and e-cigarette use (11.5%) [32]. This study investigated how smoke-free ordinances and policies impact the use of each of these products.

## 2. Materials and Methods

### 2.1. Design

Data from the 2017 Mississippi Youth Tobacco Survey (YTS) were used in this study. The Center for Disease Control’s Office on Smoking and Health developed the methodology and the core content of the Mississippi Youth Tobacco Survey. The authors developed the supplemental survey items. 

The Mississippi YTS was administered to public high school students in the fall of 2017 (*n* = 1923). All regular public schools in Mississippi containing at least one grade between 9th and 12th were included in the sampling frame obtained from the Mississippi State Department of Education. The Research Triangle Institute (RTI) applied a dual-stage cluster sample design to produce a representative sample of students. In the first stage, public high schools were selected with a probability proportional to the enrollment size. In the second stage, classrooms were chosen based on systematic equal probability sampling within each school and all students in selected classes were eligible to participate. Each participating school received student surveys, an administration guide, pencils, and parental waiver of consent forms. Teachers administered the anonymous surveys to students during class time. The Institutional Review Board for the Protection of Human Subjects in Research (IRB) at Mississippi State University approved the current study; Approval number 16-176. 

### 2.2. Sample Processing and Weights

- All completed surveys were sent to RTI for processing and weighting of data. A weighting factor was applied to each student record to adjust for non-response at the school, class, and student level. Weight = W_1_ × W_2_ × f_1_ × f_2_ × f_3_ × f_4_
- W_1_ = inverse of the probability of selecting the school
- W_2_ = inverse of the probability of selecting the classroom within the school
- f_1_ = a school-level non-response adjustment factor calculated by school size (small, medium, large)
- f_2_ = a class adjustment factor calculated by school
- f_3_ = a student-level non-response adjustment factor calculated by class
- f_4_ = a post stratification adjustment factor calculated by gender and grade

### 2.3. Measures

#### 2.3.1. Smoke-Free Ordinances and Household Rules

Although Mississippi has not enacted a statewide smoke-free ordinance, 151 municipalities had implemented local smoke-free ordinances at the time of data collection. Mississippi Tobacco Data provides a summary of Mississippi municipalities with smoke-free ordinances [33]. Students who attended high school in a municipality with a smoke-free ordinance were compared to those whose high school was not in a municipality with a smoke-free ordinance in the fall of 2017.

Students were asked, “Inside your home (not including decks, garages, or porches) is smoking always allowed, allowed only at certain times or in some places, or never allowed”? Those who reported “never allowed” were considered to live in a smoke-free home.

#### 2.3.2. Demographic Characteristics and Covariates

Students self-reported gender, age, grade in school, and race. Three categories for race were applied: white, African-American, and other. Students also responded to “Does anyone who lives with you now smoke cigarettes?” Those who reported yes were categorized as living with a smoker. We included this variable as a covariate, given that prior research illustrated that living with a smoker was a strong predictor of youth smoking initiation and past 30-day use [34].

#### 2.3.3. Secondhand Smoke Exposure and Tobacco Use

Students were asked, “In the past 7 days, did someone smoke tobacco products in your home while you were there?”, “did you ride in a vehicle where someone was smoking?”, “did you breathe the smoke from someone who was smoking tobacco in an indoor public place?”, “did you breathe in the smoke from someone who was smoking tobacco in the place where you work?”, and “did you breathe the smoke from someone who was smoking tobacco at your school?” Students who reported yes to any of these questions were considered to have past 7-day exposure to tobacco smoke.

Students were asked, “During the past 30 days, on how many days did you smoke cigarettes?”, “did you smoke cigars, cigarillos, or little cigars?”, “did you use chewing tobacco, snuff, or dip”, and “did you use e-cigarettes?” Students who did not report 0 days, were considered to be a past 30-day user of that product. 

### 2.4. Statistical Analyses

Statistical analyses were conducted using SPSS 22.0 with complex sampling procedures. Chi-square analyses compared past 7-day exposure to SHS and past 30-day tobacco use among students who lived in municipalities with a smoke-free ordinance and those that did not as well as those who lived in homes with and without smoke-free policies. Logistic regression models examined the relationship of SHS exposure and tobacco use with both smoke-free polices in multivariable analyses, adjusting for living with a smoker, gender, race, and grade.

## 3. Results

### 3.1. Response Rate

Among the schools sampled, 42 out of 50 schools participated (84.0%). Among students in the classes sampled, 1923 of 2174 students completed usable surveys (88.5%). The final response rate was 74.3%.

### 3.2. Sample Characteristics

Unweighted and weighted sample characteristics are presented in Table 1. The sample comprised of an almost equal number of males and females. Sample weights adjusted for under-representation of 11th graders and African-Americans. Approximately two-thirds of students attended a high school in a municipality with a smoke-free ordinance, 80%of students lived in a home where smoking was not allowed, and slightly less than half reported past 7-day exposure to secondhand smoke. Fewer than one in 10 students reported past 30-day use of cigarettes, cigars, or smokeless tobacco; whereas more than 10% reported past 30-day e-cigarette use.

### 3.3. Relationship of Smoke-Free Ordinances and Policies with Secondhand Smoke Exposure and Tobacco Use

Youth who lived in municipalities with smoke-free ordinances were less likely to report any past 7-day exposure to second tobacco smoke than those who did not; and were also less likely to report past 30-day cigarette smoking and cigar smoking (see Table 2). However, no association with smoke-free ordinances and non-combustible tobacco use was detected. Youth were equally likely to report past 30-day SLT and e-cigarette use.

Youth who lived in homes with smoke-free policies were less likely to report any past 7-day exposure to secondhand tobacco smoke than those who did not; and were also less likely to report past 30-day use of both combustible and non-combustible tobacco products (see Table 3).

Although both local smoke-free ordinances and household policies influenced SHS exposure and at least some forms of tobacco use, household policies appear to have a greater impact than local ordinances in multivariable analyses (see Table 4). In logistic regression models including local ordinances and household policies, as well as gender, race, and grade; youth who did not live in smoke-free homes were more likely to be exposed to SHS and use tobacco products than those who lived in smoke-free homes. Local smoke-free ordinances were not associated with these outcome measures in multivariable analyses. 

## 4. Discussion

Local smoke-free ordinances and household policies were associated with lower rates of SHS exposure and cigarette smoking among youth. These results are consistent with previous research on youth and smoking policies [29,30,31]. To our knowledge, this was one of the first studies to examine whether or not these restrictions also protected youth against other forms of tobacco use. Household smoke-free policies were also protective against cigar, SLT, and e-cigarette use. Although, local smoke-free ordinances were also protective against cigar use, they were not associated with non-combustible use. Youth who lived in places with a smoke-free ordinance were no less likely to use SLT or e-cigarettes than those who were not protected by a local ordinance.

The results also suggested that household policies had a stronger influence on SHS exposure and tobacco use than for smoke-free ordinances. Associations in bivariate analyses were stronger and more consistent for smoke-free home policies than local ordinances; and in multivariable analyses, smoke-free home policies remained significant predictors of SHS exposure and tobacco use, whereas local policies did not.

Our data cannot directly explain why household policies had a stronger association with SHS exposure and tobacco use. Perhaps youth spent more time in their homes than in restaurants and workplaces, or perhaps youth were more impacted by household norms than community norms. 

Household members have more direct control over rules for their homes than for their municipality, and thus decisions to prohibit smoking may have a greater impact on household youth than local ordinances. Results clearly demonstrated that both of these smoke-free policies were protective against SHS exposure and combustible tobacco use. Future research should investigate why local ordinances have a weaker impact on SHS exposure and combustible tobacco use, and why there was no relationship with non-combustible tobacco use.

There are potential limitations to this study. The sample was limited to youth in Mississippi public schools and may not be generalized to other populations. Furthermore, youth self-reported SHS exposure and tobacco use, and these reports were not biologically verified. 

## 5. Conclusions

The results suggested that smoke-free ordinances and policies offer protection against exposure to tobacco smoke and the use of combustible tobacco products However, smoke-free ordinances and policies did not appear to impact on non-combustible tobacco use such as through smokeless tobacco and e-cigarettes. Tobacco-free, rather than smoke-free ordinances might offer more protection for youth.

## Figures and Tables

**Table 1:** Sample characteristics.

Characteristic	Unweighted Sample Size	Unweighted Percent	Weighted Percent
Gender: Male	967	50.90%	49.80%
Female	934	49.10%	50.20%
Age: 13	11	0.60%	0.50%
14	481	25.00%	19.40%
15	510	26.50%	26.90%
16	323	16.80%	22.80%
17	455	23.70%	24.10%
18	116	6.00%	5.60%
19+	10	0.50%	0.40%
Grade: 9th	685	35.90%	27.20%
10th	436	22.80%	25.80%
11th	298	15.60%	24.00%
12th	491	25.70%	23.00%
Race: White	976	51.60%	41.80%
AA	715	37.80%	48.70%
Other	200	10.60%	9.50%
Past 7-day SHS exposure: Yes	880	47.30%	45.20%
No	982	52.70%	54.80%
Past 30-day cigarette use: Yes	146	8.10%	7.20%
No	1664	91.90%	92.80%
Past 30-day cigar use: Yes	163	8.70%	8.40%
No	1704	91.30%	91.60%
Past 30-day SLT use: Yes	139	7.50%	6.60%
No	1721	92.50%	93.40%
Past 30-day e-cigarette use: Yes	237	12.70%	11.50%
No	1629	87.30%	88.50%
Smoke-free ordinance: Yes	1256	65.30%	66.40%
No	667	34.70%	33.60%
Home is smoke-free: Yes	1473	80.00%	80.70%
No	368	20.00%	19.30%
Smoker in the home: Yes	583	30.30%	29.20%
No	1340	69.70%	70.80%

**Table 2:** SHS exposure and tobacco use by smoke-free ordinance status.

Outcome	Smoke-Free Ordinance	No Ordinance	*p*
Past 7-Day SHS Exposure	41.90%	51.50%	0.04
Past 30-Day Cigarette Use	5.10%	11.40%	0.003
Past 30-Day Cigar Use	7.20%	10.90%	0.04
Past 30-Day SLT Use	6.60%	6.50%	ns
Past 30-Day E-Cigarette Use	11.20%	12.10%	ns

**Table 3:** SHS exposure and tobacco use by smoke-free home status.

Outcome	Home is Smoke-Free	Home is Not Smoke-Free	*p*
Past 7-Day SHS Exposure	38.00%	74.60%	<0.001
Past 30-Day Cigarette Use	4.80%	17.60%	<0.001
Past 30-Day Cigar Use	6.40%	16.40%	<0.001
Past 30-Day SLT Use	4.90%	13.20%	<0.001
Past 30-Day E-Cigarette Use	9.60%	19.50%	<0.001

**Table 4:** Logistic regression of SHS exposure and tobacco use on smoke-free policies ^1^.

Outcome	No Smoke-Free Ordinance	Home is Not Smoke-Free
	OR (95% CI)	OR (95% CI)
Past 7-Day SHS Exposure	1.1 (0.9–1.3)	4.3 (3.1–6.1)
Past 30-Day Cigarette Use	1.2 (0.8–1.7)	3.7 (2.3–6.0)
Past 30-Day Cigar Use	1.4 (0.8–2.4)	2.6 (1.5–4.4)
Past 30-Day SLT Use	0.6 (0.4–1.0)	2.6 (1.6–4.2)
Past 30-Day E-Cigarette Use	0.7 (0.4–1.3)	2.1 (1.5–2.9)

^1^ Adjusted for smoker in the home, gender, race, and grade.
